# Enhancing microstructure and mechanical properties of nickel aluminium bronze alloy through tin addition

**DOI:** 10.1038/s41598-023-44146-y

**Published:** 2023-10-07

**Authors:** Sushanth Poojary, Vikas Marakini, Rajath N. Rao, Vijeesh Vijayan

**Affiliations:** Department of Mechanical Engineering, Nitte (Deemed to Be University), NMAM Institute of Technology (NMAMIT), Nitte, 574110 India

**Keywords:** Engineering, Materials science

## Abstract

This article describes the changes in the microstructure, cooling curve characteristics and mechanical properties of cast Nickel Aluminium Bronze alloy (NAB) alloy that were produced by the addition of various amounts of Tin (Sn). The solidification parameters were recorded using a computer-aided cooling curve analysis setup, and optical and scanning electron microscopes were utilised to study the evolution of the microstructure. The chemical composition of different phases generated in the NAB alloy with and without Tin was investigated using an X-ray diffraction technique. With the addition of tin, the alloy's microstructure changed from columnar to equiaxed grain structures, and the ideal microstructure was produced at a Tin concentration of roughly 1.0 weight percent. The formation of the high temperature α and the grain boundary Sn rich phases across the alloy microstructure as a result of further addition has a considerable impact on the alloy's increased hardness (upto 69%) and tensile strength (upto 28.4%) compared to untreated NAB alloy. Influence of Sn on microstructure transformation is confirmed by the decline in alloy nucleation temperatures, the reduction in undercooling intensity, and the decrease in cooling rate during solidification. The addition of Tin to the NAB alloy caused morphological changes in the kappa (K) phases, which are also reported in the this article. In addition to this, the research makes an attempt to describe the mechanism underlying the formation of equiaxed grains and phase transformations in Sn-treated NAB alloys.

## Introduction

NAB, or nickel-aluminium-bronze, is a copper-zinc alloy that typically contains 9 to 12% Al, as much as 5% Fe, and 3% Ni. The ease of casting and high strength of these alloys have made them ideal for applications such as maritime propellers, valves, pump blades, and more^1,2^. The microstructure and micro constituents of NAB materials have a major impact on their characteristics^3^. Copper-rich phase α, β’ phases (martensitic), and multiple intermetallic Kappa phases (K_I_, K_II_, K_III_, and K_IV_) make up the typical microstructure of a NAB alloy. Phases K_I_, K_II_, K_III_, and K_IV_ indicate rosette, globular, lamellar, and fine globular form in intermetallic Kappa phases, respectively^4^. Even at equilibrium cooling rates, the NAB alloy goes through a number of changes to its microstructure. The liquid first turns into a single-phase β structure when it solidifies, and then, as the material cools furthermore, α phase precipitates into grains from the borders, creating a Widmanstatten structure. The iron-rich K_I_ phase would precipitate out of the rosette shape, if it were cooled even more. Moreover, K_II_ and K_III_ phases are generated from inside grains at lower temperatures. Lastly, tiny K_IV_ phase particles precipitate^5,6^.

However, despite their versatile utility, NAB alloys often succumb to failure mechanisms, including erosion, erosion-corrosion, and cavitation, primarily rooted in the non-uniform development of microstructure phases during solidification. Erosion-corrosion arises from the non-uniform development of microstructural phases during solidification. Surface treatment or modification and the addition of dopants are two approaches that may be used to boost the corrosion resistance and mechanical properties of NAB alloy. One innovative technique for enhancing erosion-corrosion resistance involves the application of the friction stir technique with the addition of chromium reinforcement (FSP)^7^. Additionally, studies have demonstrated the potential to mitigate failure by refining K_I_ and K_II_ phases through the incorporation of elements such as C_e_, S_m_, and Y_b_ into cast NAB alloys^8^. This refinement is particularly beneficial in reducing crack propagation along coarser K_I_ and K_II_ phase boundaries. Furthermore, the introduction of titanium as a dopant has shown promise, leading to improved mechanical characteristics and the formation of finer grains in NAB alloys^9^. Notably, the addition of tin to brass has resulted in the emergence of a new phase, thereby enhancing the alloy's hardness through microstructural alterations^10^. Extending beyond these advancements, the incorporation of tin and nickel into copper alloys has demonstrated enhanced corrosion resistance and improved material properties^11^. Tin, in particular, has been found to be a potent enhancer of both corrosion resistance and material characteristics. Finally, research into fretting corrosion within tin-plated copper alloys has highlighted temperature as a critical influencing factor^12^. The exceptional corrosion resistance exhibited by Sn bronzes, a copper-tin alloy, further underscores the significance of Sn as an alloying element. While Sn is added to copper to preserve its structural integrity, it should be noted that its solubility in copper at room temperature is limited.

Recent research has shown that virtual simulation may help optimise design by providing insight into melt flow and casting solidification, which in turn reduces casting defects and losses^13^. Nevertheless, the quality of these simulated models is dependent on input of temperature dependent features of the mould, which limits their applicability. The thermo-physical characteristics of the alloy may be efficiently deduced with the use of computer-aided cooling curve analysis (CACCA), which can also be used to learn more about the phase transition temperatures of the individual alloy components. Using the captured cooling curve, one may ascertain the liquidus temperature, the degree of recalescence of the solidus temperature, and undercooling, all of which are essential aspects of solidification^14^. Nevertheless, the chemical composition and cooling rate greatly influence the thermal properties and cooling rate during solidification^15^. Thermal treatments influences the microstructure, mechanical characteristics by increasing the erosion-corrosion resistance with increase in α-phase.

In response to these challenges, this study embarks on a novel exploration, which is the influence of tin content on NAB alloy. By systematically varying the Sn content in the alloy, this study seek to unravel an uncharted territory by shedding light on how this addition impacts the alloy's cooling curve characteristics, microstructure, and hardness. The primary objective is to identify innovative ways to tailor the properties of NAB alloys, potentially enhancing their suitability for a wide spectrum of applications. Through a detailed examination of microstructural changes, nucleation temperatures, and mechanical properties induced by Sn, this study aim to provide valuable insights that can guide the design and development of advanced NAB alloys, setting new benchmarks for performance and durability.

## Material & methods

**Figure 1:**
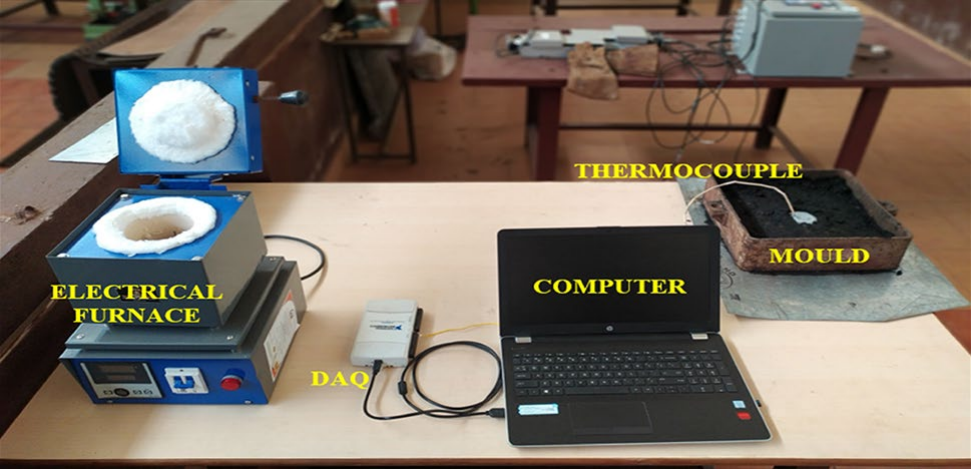
Photograph image of the CACCA set-up used to record the solidification history of the alloy sample.

The study used NAB alloy having (Cu-78.31%, Al-10.92%, Ni-4.86%, Fe-3.99%, Mn-1.31%, Sn-0.23%, Balance-0.33%). Around 800 g of alloy cut from the ingot was melted using a graphite crucible heated to about 1200 °C in an electric resistance boiler with a graphite crucible. Commercially pure tin was added to the molten metal at varied weight percentages of 0.5%, 1%, 1.5%, 2%, and 2.5%, respectively, of the ingot taken. Following 15 min of incubation, the liquid metal was poured into a cylindrical sand mould of 40 mm in diameter and 40 mm in length, which was fitted with a K-type thermocouple, as shown in Fig. 1. A coverall flux (CUPRAL) was used to protect the melt from oxide formation. The dross was later skimmed off from the melt surface prior to the pouring. Besides, the melting experiments were carried in a closed lid furnace on dry sunny days when the relative humidity was less than 60%. Furthermore, the inclusion of sand and slag inclusions were prevented by carefully skimming the dross and pouring to the newly prepared sand mould with sprue.

The temperature history of the solidifying melt was recorded at 100 Hz using a computer connected National Instrument DAQ-6210 Express module. The solidified alloy's thermal properties were analysed by plotting the collected temperature data. For metallographic examination, samples were cut from the solidified piece and polished with papers of 80 to 1500 grit, followed by polishing with 1 m diamond paste on velvet cloth. The samples are then etched for approximately 10 s with a freshly prepared Ferric-Chloride solution. Using a Leica DM750M optical microscope, the microstructure of untreated and treated materials is captured. The samples are afterwards examined using a Scanning Electron Microscope (SEM)—ZEISS SEM EVO18. The microhardness of the samples are measured using Mitutoyo HM-200 Micro-Vickers hardness tester under a 100 gf load for 15 s. Tensile specimens are fabricated in accordance with the ASTM E8 standard, featuring dimensions of 40 × 13 × 4mm. Subsequently, the tensile testing procedure is conducted utilizing the Shimadzu tensile testing apparatus. Here, five readings of hardness and tensile strength are taken for each condition and the mean values are calculated.

## Results and discussion

### Formation of columnar structure in untreated NAB alloy

**Figure 2:**
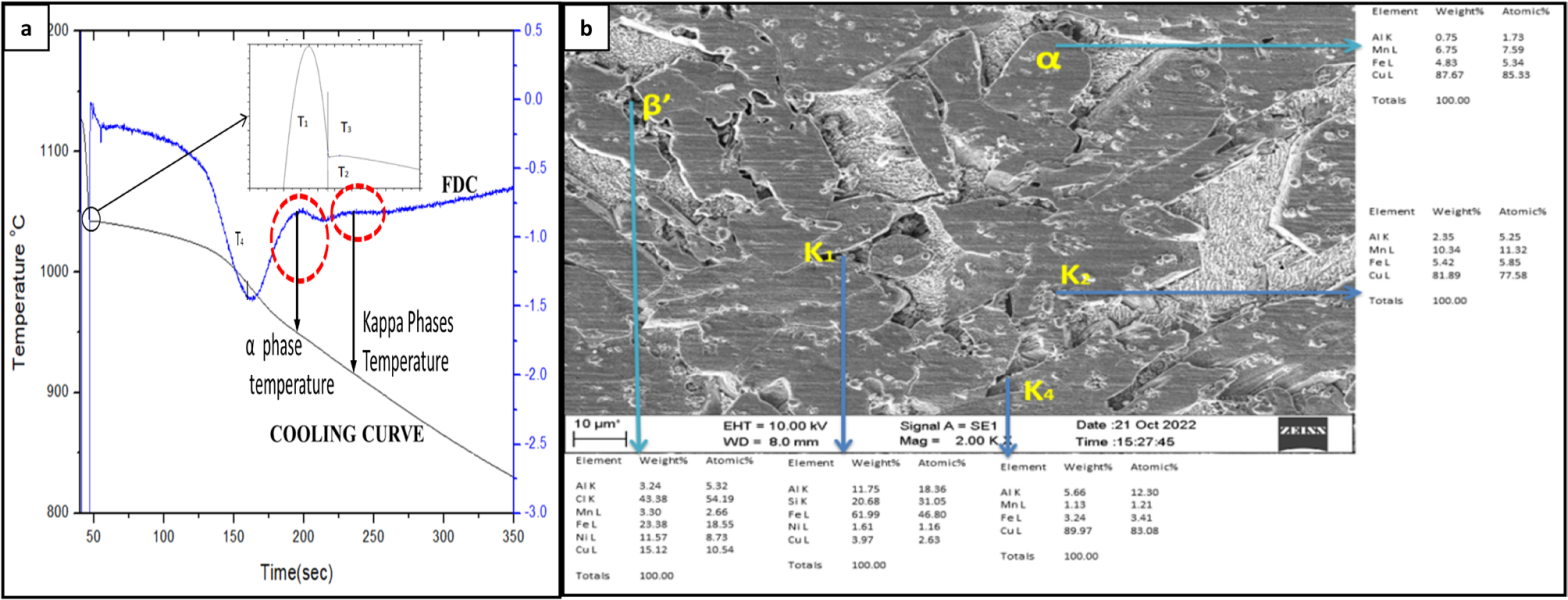
(**a**) Cooling curve and first derivative curve of untreated NAB alloy, (**b**) SEM microstructure of untreated NAB alloy, indicating α, β’ and different K phases.

Figure 2a depicts the cooling curve of the untreated alloy, and Table 1 lists the nucleation temperatures. The untreated alloy cooled at a rate of 0.71 °C/s during solidification showed four temperature characteristics related to solidification, which can be determined from the cooling curve: T1: nucleation start temperature, T2: minimum nucleation temperature, at which a stable nucleus has formed in the liquid, T3: temperature at which growth of the formed nuclei begins, and T4: solidification completion temperature, as shown in Fig. 2a. Additional nucleation factors, such as degree of undercooling and recalescence temperature must be obtained from temperatures T1, T2, and T3 accordingly.

**Table 1:** Solidification parameters of NAB alloys obtained using CACCA.

Condition	T1 ℃	T2 ℃	T3 ℃	T4 ℃	Undercooling temperature t_1_-t_2_ ℃	Recalesence temperature t_3_-t_2_ ℃	Cooling rate ℃/sec
Untreated	1045.4	1038.4	1038.9	984.4	7	0.5	0.71
0.5%Sn	1040.4	1033.6	1033.9	951.4	6.8	0.3	0.7
1.0%Sn	1036.8	1030.1	1030.3	928.5	6.7	0.2	0.69
1.5%Sn	1029.6	1023.0	1023.0	966	6.6	0	0.68
2.0%Sn	1022.8	1016.3	1016.3	939.5	6.5	0	0.66
2.5%Sn	1017.2	1010.8	1010.8	912	6.4	0	0.63

Based on the CACCA results, it shall be deduced that the nucleation of the untreated alloy, preferably β phase begins at about 1045.4 ℃ with an undercooling of about 7 ℃ and this liquid to solid transformation is completed at about 1038 ℃. Soon after, the β phase undergoes a solid state transformation into α phase initially, and then further into kappa phases as indicated in the cooling curve shown in the Fig. 2a. This solid phase transformation of β is well reported in literature^16^. Subsequently, Fig. 2b shows the SEM microstructure obtained in the present study of untreated NAB alloy, which depicts a columnar structure of α-phase, the martensitic β’-phase (black etching constituent) and Kappa phases such as K_I_, K_II_, K_III_ and K_IV_.

### Effect of Sn addition on the cooling curve parameters and the microstructure of NAB alloy

**Figure 3:**
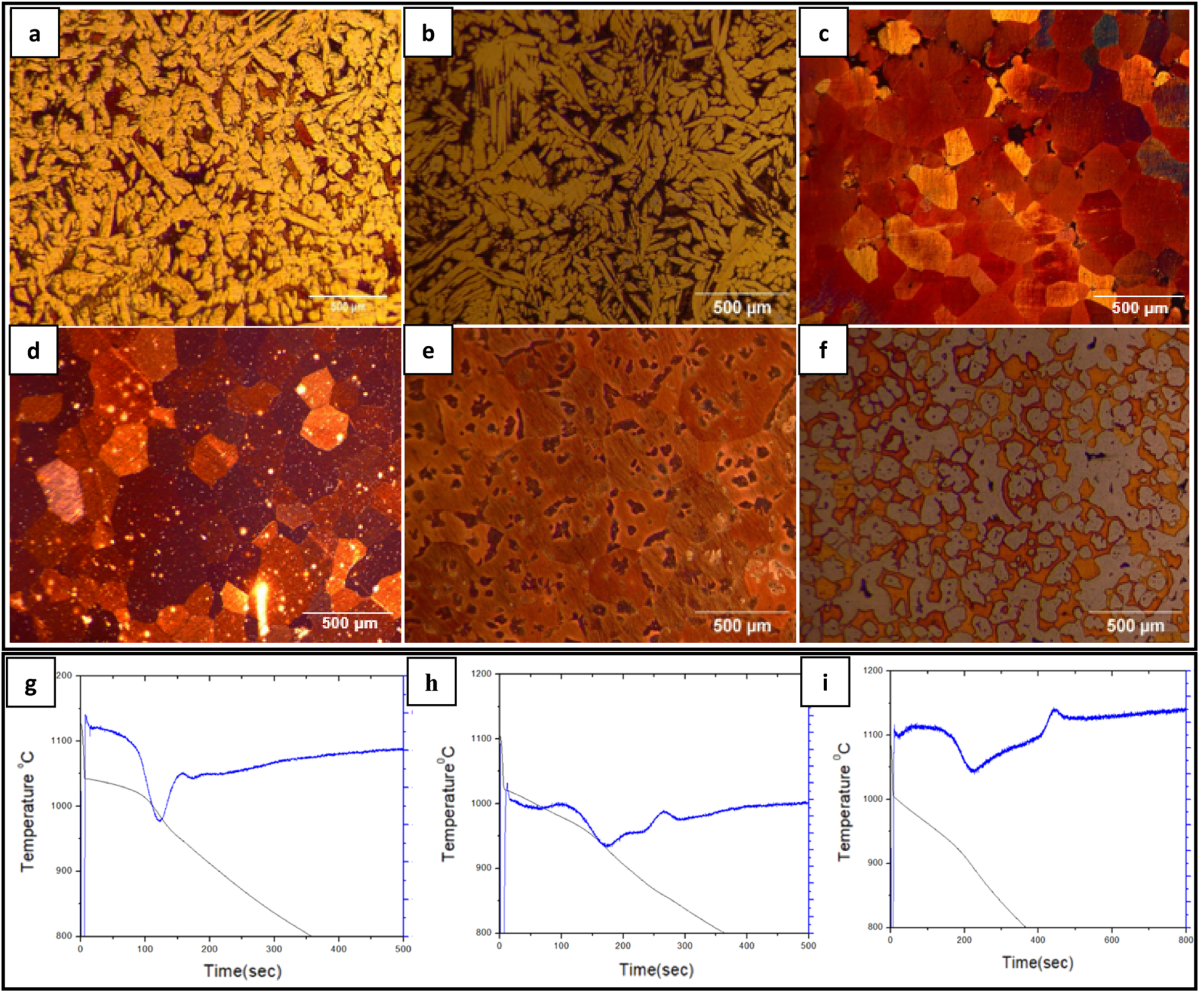
Microstructure of NAB with addition of Sn (**a**) 0%, (**b**) 0.5%, (**c**) 1%, (**d**) 1.5%, (**e**) 2%, (**f**) 2.5%, (**g**) Cooling curve of untreated NAB, (**h**) 1wt% Sn and (**i**) 2wt% Sn alloy.

Figure 3a–f shows the impact of increasing quantities of Sn addition on the microstructure of NAB alloy. As noted, the addition of Sn has resulted in a change in the microstructure of the NAB alloy; the untreated NAB alloy, which exhibited a columnar microstructure, was finally changed into an equiaxed structure as the Sn concentration increased. Amongst, at 1 wt.% addition of Sn, the NAB alloy showed fully equiaxed grains compared to untreated NAB alloy. However, further increment in wt.% addition of Sn resulted in the formation of high temperature α’-phase of copper alongside the equiaxed grains of room temperature stable α phase.

Table 1 shows the changes observed in the cooling temperature characteristics as the percentage of Sn added to the alloy is increased. With increased Sn addition, the nucleation temperatures (T1, T2, T3, and T4), degree of undercooling, and recalescence temperature related with the nucleation of phase has decreased. The decrease in undercooling with the addition, indicates that Sn is favouring the nucleation of β phase from liquid, either by providing heterogeneous nucleation sites or by decreasing the activation energy required for the nucleation^14^. And the drop in nucleation temperatures was a result of Sn's solubility and subsequent alloying^15^. On the other hand, the lowering recalescence temperature suggests that the latent heat generated by freshly created stable nuclei decreases with increasing Sn concentration; nevertheless, above 1.5 wt% Sn, the latent heat cannot be detected using the current apparatus. However, similar behaviour is found during heterogeneous nucleation and when nucleation occurs concurrently in many areas, resulting in a tiny and symmetrical structure^17^.

### Effect of Sn addition on the solid state transformation temperatures

**Figure 4:**
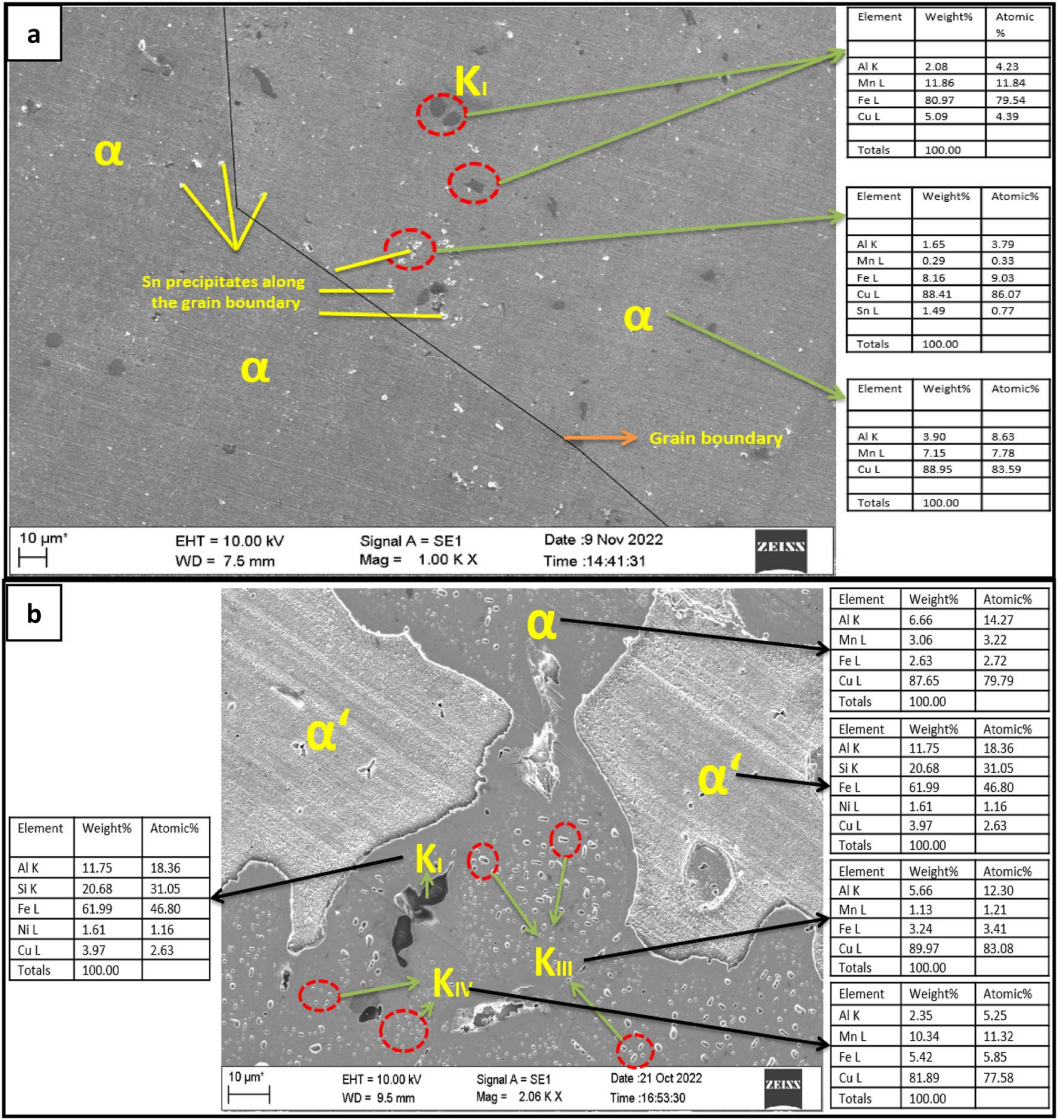
Energy dispersive X-ray metallographic image of (**a**) NAB with 1.5% Sn, (**b**) NAB with 2.5% Sn.

The addition of Sn has resulted in the suppression of the peak observed in the FDC curve of untreated NAB alloy, a peak formed due to the transformation of β-phase into Widmanstaten α & β, shown in the Fig. 3g–i. Instead, two additional peaks were observed in the cooling and FDC curves shown in Fig. 3h, one prior to T4 temperature at about 980℃ and another peak at about 860℃. The former peak would be due to the transformation of β to high temperature α’ (irregular morphology) and β (metastable and equiaxed phase), indicated in Fig. 4a and the later peak would be due to the transformation of the β (metastable and equiaxed) into low temperature α.

### Effect of Sn addition on kappa phases

Figure 2b shows the microstructure of an untreated alloy, which has all the kappa phases described in the literature. However, the kappa phases observed in the Sn-treated alloy are unique from those shown in the NAB, since the addition of Sn has diminished the usual solid state transition processes observed in the NAB. Figure 4a depicts the fine K_I_ phase with a butterfly shape that formed in the 1wt%Sn-treated alloy, whereas Fig. 4b depicts the kappa phases that formed in the 2.5wt%Sn-treated alloy. In all scenarios, however, the K_II_ phase was not observed.

### Effect of Sn addition on the mechanical properties

**Figure 5:**
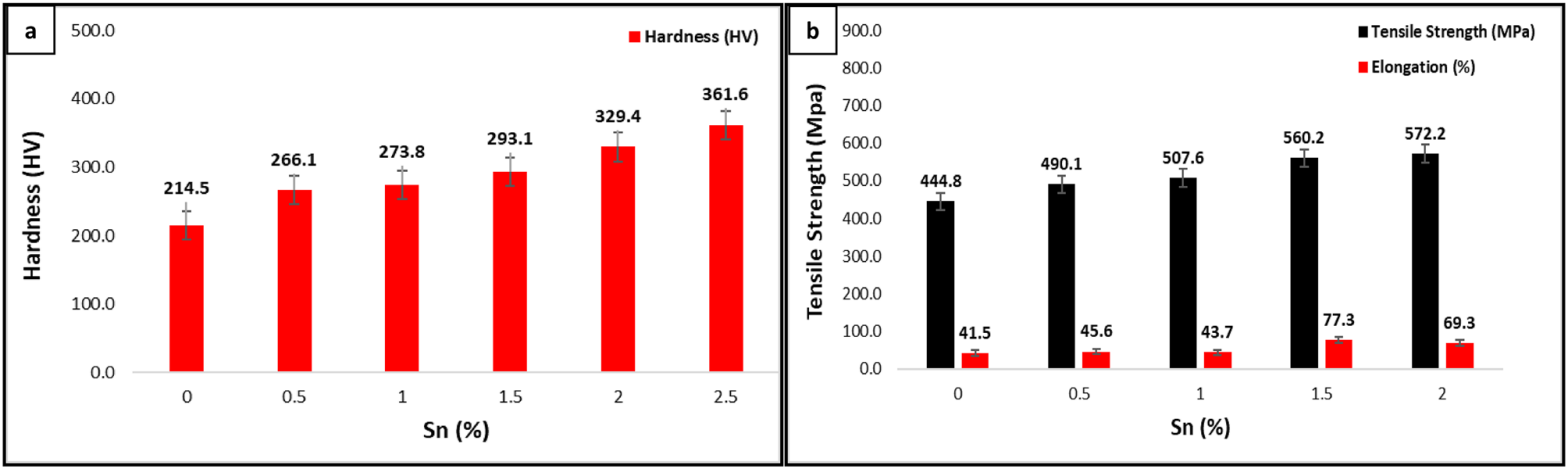
(**a**) Hardness value of NAB alloy with addition of Sn, (**b**) Tensile strength of NAB alloy with addition of Sn.

The results of hardness testing for both the untreated alloy and the alloy treated with Sn are shown in Fig. 5a. Similarly, Fig. 5b displays the outcomes related to tensile strength and elongation for these two alloy variants. The increased tensile strength and increased alloy hardness observed in the Sn-treated samples can be attributed to transformations in microstructure and alterations in the kappa phases. Notably, the addition of Sn, ranging from 0 to 2.5%, led to a hardness range of 214 HV to 361.63 HV, and a parallel variation in tensile strength from 444.8 to 572.2 MPa.

The intriguing observation depicted in Fig. 5b, suggesting a direct relationship between tensile strength and ductility within the studied alloys, warrants a deeper exploration. Although it is conventionally expected that increasing tensile strength tends to come at the expense of reduced ductility, exceptions to this rule do exist, as demonstrated here. Several key factors contribute to this phenomenon. Firstly, the observed interplay between tensile strength and ductility is closely linked to the microstructural alterations induced by the introduction of tin (Sn) into NAB alloys. These alterations encompass changes in grain structure, distribution of phases, and the presence and morphology of intermetallic kappa phases, collectively influencing both tensile strength and ductility. Additionally, the transition from a columnar to equiaxed grain structure, facilitated by Sn addition, can strengthen both tensile strength and elongation, a trend previously observed in modified Al-Si alloys^18^. In such alloys, the transformation of acicular eutectic silicon into a fine fibrous structure, achieved through modification with elements like Na, Sr, or Ca, results in enhanced tensile properties. Sn, serving as an alloying element, introduces complexity to the microstructure, its impact varying with concentration and overall alloy composition. Furthermore, the potential induction of phase transformations by Sn, leading to the formation of specific phases such as α' and β'', may contribute to this distinctive and noteworthy relationship between tensile strength and ductility.

Comparing the Energy Dispersive X-ray Spectroscopy (EDAX) outcomes for the untreated and Sn-treated alloys as demonstrated in Figs. 2b and 4a, it becomes evident that changes in the α phase's Fe content contribute to these alterations. Furthermore, the existence of phases enriched in Sn at the grain boundaries, coupled with the development of finely equiaxed grain structures, emerge as the central factors driving the observed enhancement in alloy hardness.

### Mechanism of equiaxed grain formation in Sn doped NAB alloys

**Figure 6:**
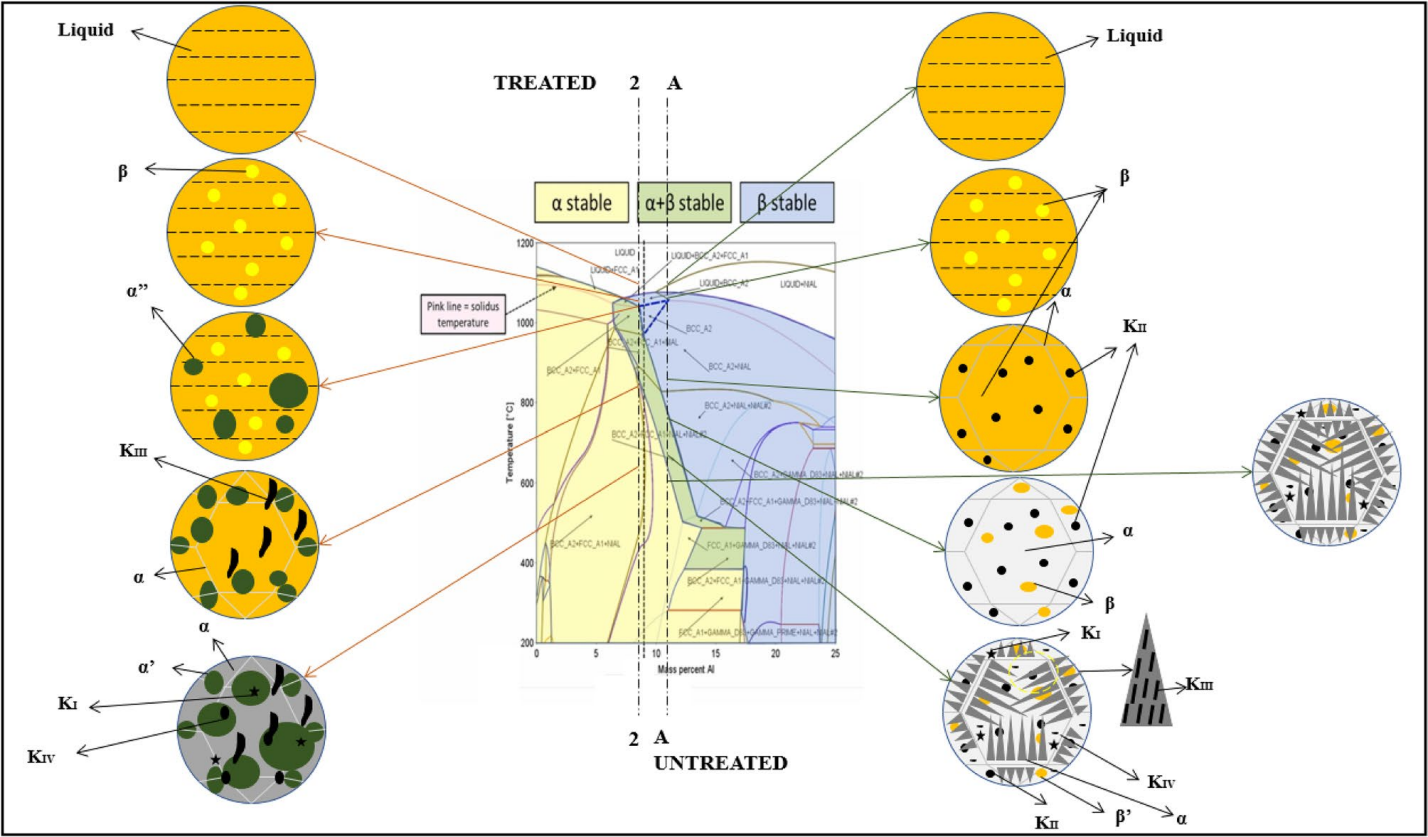
Phase diagram of Cu–XAl–4.5Ni–4Fe–1.15Mn (wt.%) alloy^16^.

The transformation observed in the microstructure of the Sn treated alloys shall be explained using Cu-XAl-4.5Ni-4Fe-1.15Ni phase diagram, reported by Orzolek et al.^16^, shown in the Fig. 6. As observed, an untreated alloy with about 11% Al would trace the line A–-A (shown in Fig. 6) during solidification. In agreement with the CACCA reported in this study, an untreated NAB alloy liquid would start the solidification by transforming into β crystals at about 1045℃ and the formed β would grow into equiaxed grains with precipitation of K_II_ phase with decrease in temperature. This transformation would be complete by about 980℃, and microstructure will contain entirely of equiaxed structure as illustrated in the Fig. 6. However, further decrease in the temperature during solidification process will result in the entry into the two phase region of the phase diagram and would result in the formation of equiaxed α and β phase, K_III_ phase will precipitated during this transformation before 820 ℃. Further decrease in the temperature would result in the growth of Widmanstaten α from the grain boundaries of equiaxed α gains until about 790℃ and the K_IV_ phase will be formed alongside. Further cooling would result in the formation of K_I_ phase at the lower temperatures. Hence, final microstructure would subsequently contain Widmanstaten α, retained β’, K_I_, K_II_, K_III_, K_IV_ phases, which is in well agreement with the microstructure reported in the present study.

On the other hand, Sn treatment has resulted in the decrease in the nominal composition of NAB alloy towards lower side, as a result, the solidification curves would shift left in the phase diagram towards lower content of Aluminium. Presumably, 1.0 wt% Sn has caused the alloy to trace the solidification path indicated by line 2–2 in the Fig. 6. As noticed, a shift towards the left of the phase diagram leads to a drop in the melting point of the alloy; similarly, CACCA data reveal a decrease in nucleation temperature as Sn concentration increases. At 1 wt. % Sn, the β crystals nucleate from liquid at about 1036 ℃ and grow into equiaxed β crystals. This transformation completes at about 1030 ℃, however further drop in temperature would result in the formation of high temperature metastable α’’ and β’’ phases from liquid and newly formed β crystals.

**Figure 7:**
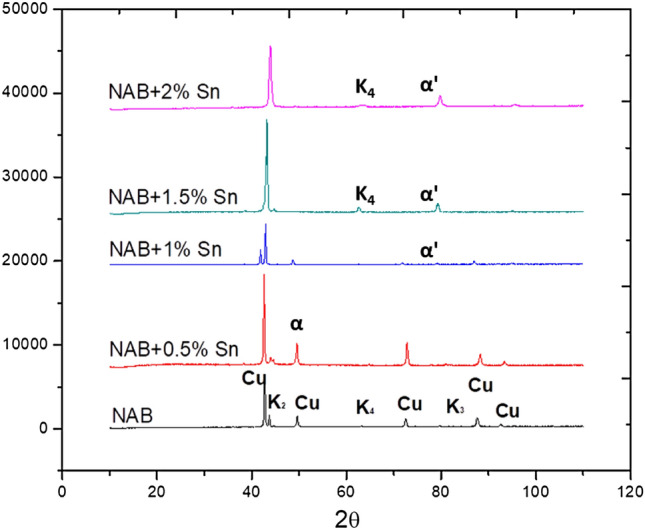
Comparison of phase constitutions of NAB alloy on addition of Sn.

Further decrease in the temperature would result in the formation of high temperature α’ and β’ phases up to 1000℃. The high temperature α’ would be precipitated towards the grain boundaries of equiaxed β phase. Further decrease in temperature would result in the formation of equiaxed α grains from β phases, also precipitation of K_III_ and K_IV_ phases. This reaction will be complete by about 820 ℃. Further decrease in the temperature below 800℃ would result in the formation of low temperature stable α phases and K_I_ in the equiaxed grains. Therefore the final microstructure of Sn treated NAB alloy at room temperature would contain α’ (formed at high temperature), room temperature α, K_I_, K_III_, K_IV_ phases. As the solidifications shifted towards left of the phase diagram, the K_II_ phase was not formed and the formation of Widmenstatten α was evaded. The X-ray diffraction (XRD) outcomes depicted in Fig. 7 corroborate the preceding discourse. Notably, the intensity of the Widmanstätten α phase diminishes with escalating Sn content. Conversely, for specimens containing 1.0 wt.% Sn and beyond, the plots reveal the emergence of novel α’ and K_IV_ phases. This concurrence aligns perfectly with the microstructural details presented in this publication.

## Conclusions

The conclusions that may be derived from the data and discussion are as follows:

1. The microstructure of the alloy changes from columnar to equiaxed with the addition of 1 weight percent or more of Sn; however, larger weight percentages of Sn addition cause the formation of an irregularly shaped high temperature α' Cu phase across the grain boundaries of the α Cu phases.
2. The reduction in the alloy's nucleation temperatures (T1, T2, T3, and T4), as well as the degree of undercooling and cooling rate, with increasing Sn concentration, indicating the impact of Sn on the nucleation and development of NAB alloy microstructure components.
3. The microstructure transformation in terms of creation of the Sn phase and changes in the kappa phases has resulted in an increase in the hardness and tensile strength and of the alloy with increase in Sn concentration.

## Data Availability

The datasets used and/or analysed during the current study available from the corresponding author on reasonable request.
